# Supplementary material for: Screening of Serum Biomarkers of Coal Workers’ Pneumoconiosis by Metabolomics Combined with Machine Learning Strategy
Source: Int J Environ Res Public Health. 2022 Jun 9;19(12):7051. doi: 10.3390/ijerph19127051 (PMC9222502; doi:10.3390/ijerph19127051)
Supplement: Supplementary file 1 [file ijerph-19-07051-s001.zip › ijerph-1735334-SI.pdf]

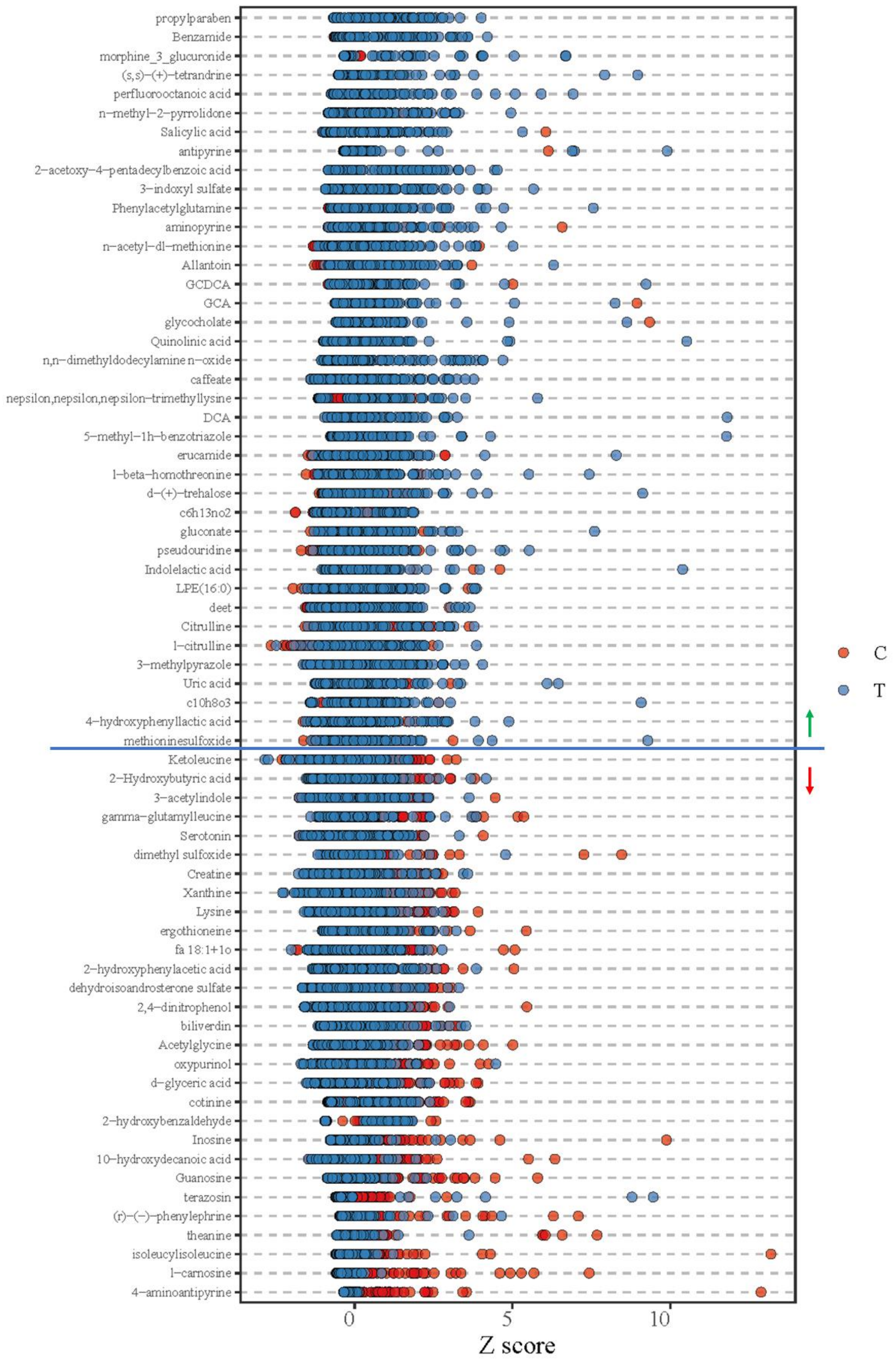

Figure S1. The relative abundance of the differential metabolites. The standard score (Z score) map was drawn to show the relative abundance of the 68 differential metabolites in the control and case groups. The metabolites above the blue line increased in the case group, and vice versa. The red point: control group (C); the blue point: CWP case group (T).

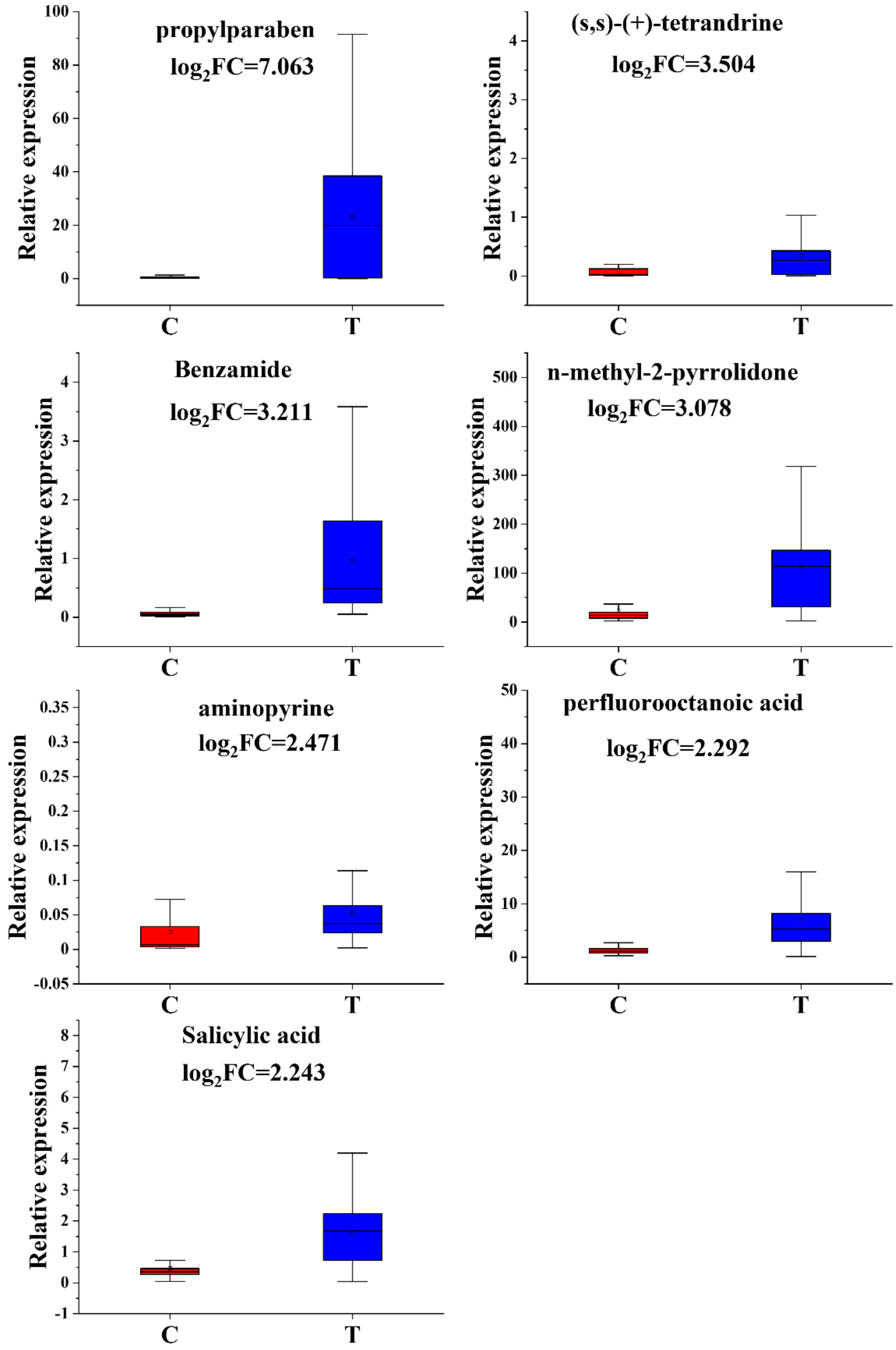

Figure S2. The top seven metabolites with the greatest difference between the control group and coal worker's pneumoconiosis (CWP) case group. According to the order of fold change (FC), they were propylparaben, (s,s)-(+)-tetrandrine, benzamide, N-methyl-2-pyrrolidone, aminopyrine, perfluorooctanoic acid and salicylic acid. The fold change (FC) of these metabolites was over 4, and their relative expression all increased in the CWP case group compared with the control group.

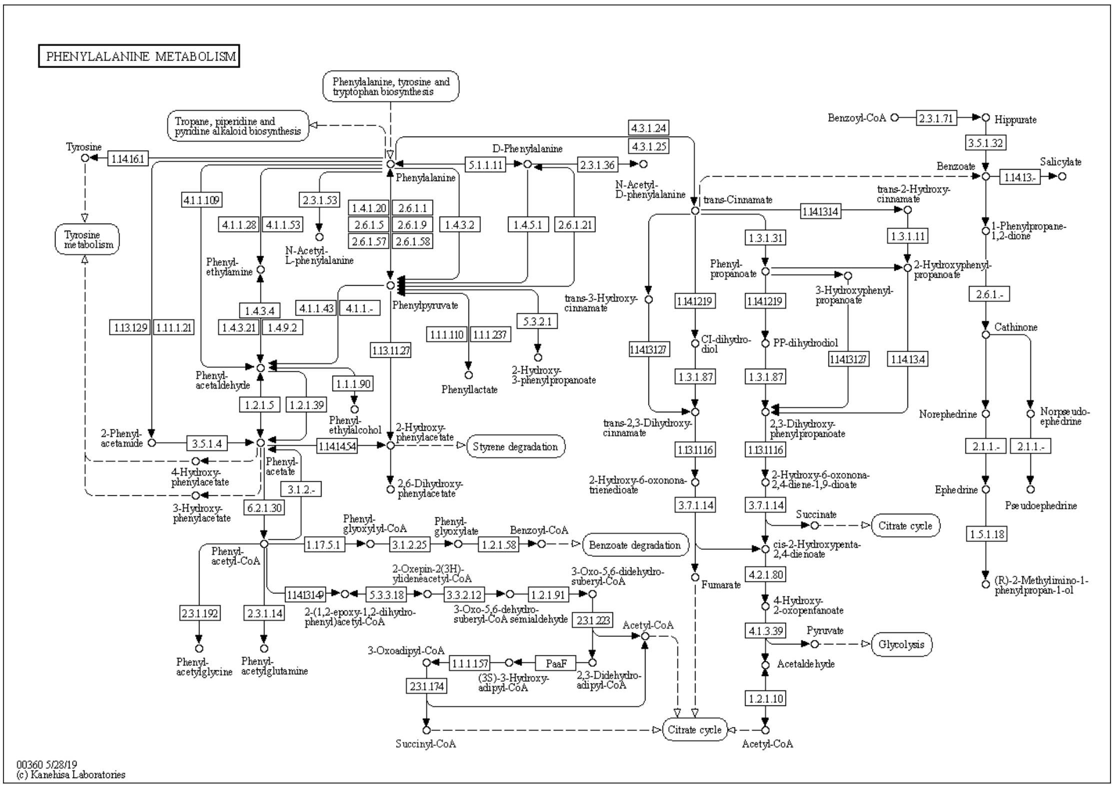

Figure S3. KEGG pathway maps of Phenylalanine metabolism.

Table S1. Differential metabolites in serum between the occupational pneumoconiosis group and the control group.

No.	Metabolite	Class	P value	FDR	$\log_2$ Fold Change	VIP
1	(r)-(-)-phenylephrine	Unknown	0.001497	0.004233	0.520	-0.943
2	(s,s)-(+)-tetrandrine	Unknown	1.37E-11	2.25E-10	11.343	3.504
3	10-hydroxydecanoic acid	Unknown	1.31E-25	6.45E-24	0.452	-1.147
4	2-acetoxy-4-pentadecylbenzoic acid	Unknown	2.98E-07	1.94E-06	1.415	0.501
5	2-hydroxybenzaldehyde	Benzene and substituted derivatives	5.02E-09	5.25E-08	0.027	-5.206
6	2-Hydroxybutyric acid	Organic Acids	7.29E-11	1.14E-09	0.790	-0.339
7	2-hydroxyphenylacetic acid	Benzene and substituted derivatives	8.57E-06	4.48E-05	0.621	-0.686
8	2,4-dinitrophenol	Unknown	2.28E-05	0.000103	0.798	-0.326
9	3-acetylindole	Unknown	0.000157	0.00059	0.726	-0.463
10	3-indoxyl sulfate	Organic sulfuric acids and derivatives	6.52E-10	7.5E-09	2.482	1.312
11	3-methylpyrazole	Unknown	7.29E-06	3.87E-05	1.190	0.250
12	4-aminoantipyrine	Unknown	1.65E-14	4.38E-13	0.203	-2.300
13	4-hydroxyphenyllactic acid	Phenylpropanoic acids	7.21E-07	4.29E-06	1.193	0.254
14	5-methyl-1h-benzotriazole	Unknown	0.016094	0.035373	1.347	0.430
15	Allantoin	Nucleotides	5.07E-26	3.5E-24	1.998	0.998
16	aminopyrine	Azoles	4.34E-15	1.5E-13	5.543	2.471
17	antipyrine	Azoles	8.6E-12	1.48E-10	2.347	1.231
18	Benzamide	Benzoic Acids	1.91E-42	6.58E-40	9.259	3.211
19	biliverdin	Tetrapyrroles and derivatives	1.51E-05	7.26E-05	0.477	-1.067
20	c10h8o3	Unknown	0.018916	0.039793	1.205	0.268
21	c6h13no2	Unknown	6.57E-08	5.04E-07	1.838	0.878
22	caffeate	Cinnamic acids and derivatives	9.4E-09	8.76E-08	1.649	0.722
23	Citrulline	Amino Acids	8.68E-07	4.99E-06	1.230	0.299
24	cotinine	Pyridines and derivatives	0.007904	0.018676	0.212	-2.240
25	Creatine	Amino Acids	4.93E-10	5.87E-09	0.721	-0.473
26	d-(+)-trehalose	Organooxygen compounds	0.000862	0.002614	1.299	0.377
27	d-glyceric acid	Organooxygen compounds	3.97E-13	9.79E-12	0.617	-0.697
28	deet	Unknown	0.001395	0.00401	1.273	0.348
29	dehydroisoandrosterone sulfate	Steroids and steroid derivatives	8.54E-07	4.99E-06	0.689	-0.537
30	DCA	Bile Acids	2.85E-05	0.000124	1.299	0.377

Table S1. Differential metabolites in serum between the occupational pneumoconiosis group and the control group. (**Continued**)

No.	Metabolite	Class	P value	FDR	log_2 Fold Change	VIP
31	dimethyl sulfoxide	Sulfoxides	6.71E-05	0.000276	0.807	-0.309
32	ergothioneine	Carboxylic acids and derivatives	0.000568	0.001865	0.636	-0.653
33	erucamide	Unknown	9.63E-13	2.08E-11	1.582	0.662
34	fa 18:1+1o	FA	3.51E-07	2.25E-06	0.735	-0.444
35	gamma-glutamylleucine	Carboxylic acids and derivatives	2.12E-08	1.88E-07	0.820	-0.287
36	gluconate	Organooxygen compounds	2.36E-05	0.000106	1.268	0.343
37	GCDCA	Bile Acids	3.54E-10	4.53E-09	2.242	1.165
38	glycocholate	Unknown	8.56E-09	8.21E-08	2.241	1.164
39	GCA	Bile Acids	2.73E-08	2.36E-07	2.463	1.301
40	Guanosine	Nucleotides	0.000637	0.002074	0.750	-0.416
41	Indolelactic acid	Indoles	3.95E-05	0.000171	1.219	0.286
42	Inosine	Nucleotides	1.1E-05	5.48E-05	0.561	-0.834
43	isoleucylisoleucine	Carboxylic acids and derivatives	2.23E-12	4.52E-11	0.470	-1.088
44	Ketoleucine	Organic Acids	3.21E-10	4.26E-09	0.832	-0.266
45	l-beta-homothreonine	Unknown	8.4E-13	1.93E-11	1.463	0.549
46	l-carnosine	Peptidomimetics	2.39E-09	2.58E-08	0.532	-0.909
47	l-citrulline	Carboxylic acids and derivatives	1.09E-09	1.21E-08	1.246	0.317
48	LPE(16:0)	LPE	5.52E-07	3.34E-06	1.325	0.406
49	Lysine	Amino Acids	7.05E-08	5.29E-07	0.791	-0.339
50	methioninesulfoxide	Unknown	3.04E-08	2.56E-07	1.249	0.321
51	morphine_3_glucuronide	Morphinans	8.07E-05	0.000328	1.477	0.562
52	n-acetyl-dl-methionine	Unknown	9.26E-19	3.99E-17	2.012	1.009
53	Acetylglycine	Amino Acids	1.49E-07	1.05E-06	0.668	-0.583
54	n-methyl-2-pyrrolidone	Unknown	1.43E-18	5.48E-17	8.444	3.078
55	n,n-dimethyldodecylamine n-oxide	Unknown	2.07E-05	9.51E-05	1.344	0.427
56	nepsilon,nepsilon,nepsilon-trimethyllysine	Unknown	5.46E-05	0.000227	1.723	0.785
57	oxypurinol	Imidazopyrimidines	5.86E-12	1.06E-10	0.650	-0.621
58	perfluorooctanoic acid	Alkyl halides	2.77E-31	3.19E-29	4.897	2.292
59	Phenylacetylglutamine	Amino Acids	2.64E-10	3.8E-09	2.167	1.116
60	propylparaben	Benzene and substituted derivatives	5.3E-15	1.66E-13	133.671	7.063

Table S1. Differential metabolites in serum between the occupational pneumoconiosis group and the control group. (**Continued**)

No.	Metabolite	Class	P value	FDR	log_2 Fold Change	VIP
61	pseudouridine	Nucleotides	3.17E-10	4.26E-09	1.221	0.288
62	Quinolinic acid	Pyridines	5.37E-08	4.31E-07	1.646	0.719
63	Salicylic acid	Benzoic Acids	2.29E-27	1.98E-25	4.733	2.243
64	Serotonin	Indoles	0.00011	0.000432	0.757	-0.401
65	terazosin	Diazinanes	3.93E-33	6.79E-31	0.172	-2.540
66	theanine	Carboxylic acids and derivatives	4.83E-08	3.97E-07	0.486	-1.039
67	Uric acid	Organic Acids	0.009546	0.022404	1.280	0.356
68	Xanthine	Nucleotides	4.04E-10	4.97E-09	0.807	-0.308
